# 2-Amino­pyrimidinium nitrate

**DOI:** 10.1107/S1600536809052362

**Published:** 2009-12-12

**Authors:** Xiao-Li Cheng, Shan Gao, Seik Weng Ng

**Affiliations:** a College of Chemistry and Materials Science, Heilongjiang University, Harbin 150080, People’s Republic of China; b Department of Chemistry, University of Malaya, 50603 Kuala Lumpur, Malaysia

## Abstract

In the title compound, C_4_H_6_N_3_^+^·NO_3_^−^, the cation is coplanar with the anion (r.m.s. deviation = 0.048 Å), and links to the anion *via* an N—H⋯O hydrogen bond, forming an ion pair. In the crystal, adjacent ion pairs are further linked by N—H⋯O hydrogen bonds into linear chains running along the *b* axis.

## Related literature

For the crystal structures of the 2-amino­pyrimidinium salts of other mineral acids, see: Czupiński *et al.* (2005 ▶); Lee *et al.* (2003 ▶); Ye *et al.* (2002 ▶).
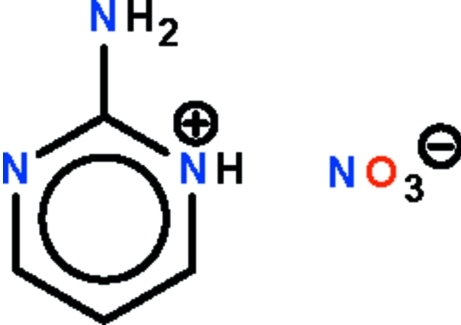


## Experimental

### 

#### Crystal data

C_4_H_6_N_3_^+^·NO_3_^−^
*M* *_r_* = 158.13
Monoclinic, 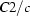

*a* = 12.632 (2) Å
*b* = 6.2160 (8) Å
*c* = 17.727 (2) Å
β = 99.009 (3)°
*V* = 1374.8 (3) Å^3^
*Z* = 8
Mo *K*α radiation
μ = 0.13 mm^−1^
*T* = 293 K
0.25 × 0.20 × 0.15 mm

#### Data collection

Rigaku R-AXIS RAPID IP diffractometer
Absorption correction: multi-scan (*ABSCOR*; Higashi, 1995 ▶) *T*_min_ = 0.968, *T*_max_ = 0.981
5139 measured reflections
1210 independent reflections
823 reflections with *I* > 2σ(*I*)
*R*_int_ = 0.028

#### Refinement

*R*[*F* ^2^ > 2σ(*F* ^2^)] = 0.039
*wR*(*F* ^2^) = 0.120
*S* = 0.99
1210 reflections
124 parameters
6 restraints
H atoms treated by a mixture of independent and constrained refinement
Δρ_max_ = 0.19 e Å^−3^
Δρ_min_ = −0.15 e Å^−3^

### 

Data collection: *RAPID-AUTO* (Rigaku, 1998 ▶); cell refinement: *RAPID-AUTO*; data reduction: *CrystalClear* (Rigaku/MSC, 2002 ▶); program(s) used to solve structure: *SHELXS97* (Sheldrick, 2008 ▶); program(s) used to refine structure: *SHELXL97* (Sheldrick, 2008 ▶); molecular graphics: *X-SEED* (Barbour, 2001 ▶); software used to prepare material for publication: *publCIF* (Westrip, 2009 ▶).

## Supplementary Material

Crystal structure: contains datablocks global, I. DOI: 10.1107/S1600536809052362/xu2705sup1.cif

Structure factors: contains datablocks I. DOI: 10.1107/S1600536809052362/xu2705Isup2.hkl

Additional supplementary materials:  crystallographic information; 3D view; checkCIF report

## Figures and Tables

**Table 1:** Hydrogen-bond geometry (Å, °)

*D*—H⋯*A*	*D*—H	H⋯*A*	*D*⋯*A*	*D*—H⋯*A*
N1—H1⋯O1^i^	0.87 (1)	1.87 (1)	2.742 (2)	177 (2)
N3—H11⋯O1	0.86 (1)	1.99 (1)	2.850 (3)	178 (2)
N3—H12⋯O2^i^	0.85 (1)	2.05 (1)	2.901 (2)	178 (2)

Symmetry code: (i) 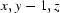.

**Fig. 1.**
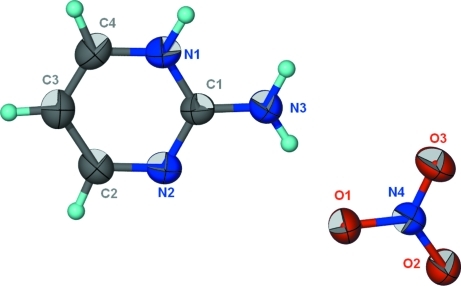
Thermal ellipsoid plot (Barbour, 2001) of [C4H6N4][NO3] at the 50% probability level; hydrogen atoms are drawn as spheres of arbitrary radius.

## supplementary crystallographic information

### Experimental

To an aqueous solution of 2-aminopyrimidine (0.19 g, 2 mmol) was added chromium nitrate nonahydrate (0.80 g, 2 mmol). The pale green solution was set aside for several days. Colorless crystals of the organic salt were isolated.

### Refinement

Carbon-bound H-atoms generated geometrically [C–H 0.93 Å, *U*(H) 1.2*U*_eq_(C)]. The nitrogen-bound H-atoms were refined with a distance restraint of N–H 0.86±0.01 Å; their temperature factors were refined.

### Crystal data

**Table:**

C_4_H_6_N_3_^+^·NO_3_^−^	*F*(000) = 656
*M**_r_* = 158.13	*D*_x_ = 1.528 Mg m^−^^3^
Monoclinic, *C*2/*c*	Mo *K*α radiation, λ = 0.71073 Å
Hall symbol: -C 2yc	Cell parameters from 3773 reflections
*a* = 12.632 (2) Å	θ = 3.3–27.5°
*b* = 6.2160 (8) Å	µ = 0.13 mm^−^^1^
*c* = 17.727 (2) Å	*T* = 293 K
β = 99.009 (3)°	Prism, colorless
*V* = 1374.8 (3) Å^3^	0.25 × 0.20 × 0.15 mm
*Z* = 8	

### Data collection

**Table:**

Rigaku R-AXIS RAPID IP diffractometer	1210 independent reflections
Radiation source: fine-focus sealed tube	823 reflections with *I* > 2σ(*I*)
graphite	*R*_int_ = 0.028
ω scan	θ_max_ = 25.0°, θ_min_ = 3.3°
Absorption correction: multi-scan (*ABSCOR*; Higashi, 1995)	*h* = −14→14
*T*_min_ = 0.968, *T*_max_ = 0.981	*k* = −7→7
5139 measured reflections	*l* = −21→20

### Refinement

**Table:**

Refinement on *F*^2^	Primary atom site location: structure-invariant direct methods
Least-squares matrix: full	Secondary atom site location: difference Fourier map
*R*[*F*^2^ > 2σ(*F*^2^)] = 0.039	Hydrogen site location: inferred from neighbouring sites
*wR*(*F*^2^) = 0.120	H atoms treated by a mixture of independent and constrained refinement
*S* = 0.99	*w* = 1/[σ^2^(*F*_o_^2^) + (0.0773*P*)^2^] where *P* = (*F*_o_^2^ + 2*F*_c_^2^)/3
1210 reflections	(Δ/σ)_max_ = 0.001
124 parameters	Δρ_max_ = 0.19 e Å^−^^3^
6 restraints	Δρ_min_ = −0.15 e Å^−^^3^

### Fractional atomic coordinates and isotropic or equivalent isotropic displacement parameters (Å^2^)

**Table:**

	*x*	*y*	*z*	*U*_iso_*/*U*_eq_	
O1	0.61627 (16)	1.0844 (3)	0.47003 (8)	0.0892 (6)	
O2	0.63012 (12)	1.3264 (2)	0.38605 (8)	0.0711 (5)	
O3	0.61420 (14)	0.9926 (3)	0.35358 (9)	0.0797 (5)	
N1	0.62478 (13)	0.3594 (3)	0.59174 (10)	0.0568 (5)	
N2	0.62700 (13)	0.7155 (3)	0.63460 (9)	0.0575 (5)	
N3	0.62544 (15)	0.6380 (3)	0.50731 (10)	0.0633 (5)	
N4	0.62009 (13)	1.1341 (3)	0.40181 (9)	0.0560 (5)	
C1	0.62639 (15)	0.5716 (3)	0.57800 (10)	0.0501 (5)	
C2	0.62721 (17)	0.6376 (4)	0.70374 (12)	0.0616 (6)	
C3	0.62702 (18)	0.4200 (4)	0.72104 (13)	0.0669 (6)	
C4	0.62560 (17)	0.2810 (4)	0.66282 (13)	0.0638 (6)	
H1	0.6222 (17)	0.268 (3)	0.5539 (10)	0.074 (7)*	
H11	0.6213 (19)	0.7733 (19)	0.4964 (16)	0.083 (8)*	
H12	0.6282 (16)	0.547 (3)	0.4718 (9)	0.068 (7)*	
H2	0.6222 (18)	0.749 (3)	0.7401 (13)	0.079 (7)*	
H3	0.628 (2)	0.378 (4)	0.7724 (10)	0.087 (7)*	
H4	0.6234 (17)	0.130 (3)	0.6655 (12)	0.068 (6)*	

### Atomic displacement parameters (Å^2^)

**Table:**

	*U*^11^	*U*^22^	*U*^33^	*U*^12^	*U*^13^	*U*^23^
O1	0.1669 (17)	0.0547 (10)	0.0505 (9)	0.0094 (10)	0.0311 (9)	0.0031 (7)
O2	0.1061 (12)	0.0523 (10)	0.0576 (9)	−0.0043 (8)	0.0217 (8)	0.0025 (7)
O3	0.1178 (13)	0.0620 (11)	0.0620 (9)	−0.0010 (9)	0.0227 (8)	−0.0182 (8)
N1	0.0695 (11)	0.0435 (11)	0.0575 (10)	0.0026 (7)	0.0104 (8)	−0.0033 (8)
N2	0.0713 (11)	0.0478 (10)	0.0543 (9)	0.0030 (8)	0.0126 (8)	−0.0039 (8)
N3	0.0928 (13)	0.0486 (13)	0.0504 (10)	0.0032 (9)	0.0174 (9)	−0.0014 (8)
N4	0.0677 (10)	0.0512 (11)	0.0506 (10)	0.0046 (8)	0.0144 (8)	−0.0020 (8)
C1	0.0535 (11)	0.0447 (12)	0.0520 (10)	0.0022 (8)	0.0077 (8)	−0.0022 (8)
C2	0.0742 (14)	0.0584 (15)	0.0533 (12)	0.0030 (10)	0.0131 (10)	−0.0047 (10)
C3	0.0787 (15)	0.0674 (15)	0.0559 (12)	0.0022 (11)	0.0146 (11)	0.0062 (12)
C4	0.0736 (14)	0.0500 (14)	0.0676 (13)	0.0011 (10)	0.0104 (11)	0.0089 (11)

### Geometric parameters (Å, °)

**Table:**

O1—N4	1.257 (2)	N3—C1	1.318 (3)
O2—N4	1.239 (2)	N3—H11	0.86 (1)
O3—N4	1.221 (2)	N3—H12	0.85 (1)
N1—C1	1.342 (2)	C2—C3	1.387 (3)
N1—C4	1.350 (3)	C2—H2	0.953 (16)
N1—H1	0.87 (1)	C3—C4	1.344 (3)
N2—C2	1.318 (3)	C3—H3	0.944 (17)
N2—C1	1.344 (2)	C4—H4	0.942 (16)
			
C1—N1—C4	121.76 (19)	N3—C1—N2	119.96 (19)
C1—N1—H1	119.8 (16)	N1—C1—N2	121.17 (18)
C4—N1—H1	118.4 (17)	N2—C2—C3	124.4 (2)
C2—N2—C1	116.65 (18)	N2—C2—H2	111.8 (15)
C1—N3—H11	120.6 (19)	C3—C2—H2	123.6 (15)
C1—N3—H12	120.0 (16)	C4—C3—C2	117.2 (2)
H11—N3—H12	119 (3)	C4—C3—H3	123.9 (16)
O3—N4—O2	122.31 (17)	C2—C3—H3	118.9 (15)
O3—N4—O1	119.30 (18)	C3—C4—N1	118.8 (2)
O2—N4—O1	118.39 (16)	C3—C4—H4	126.9 (13)
N3—C1—N1	118.86 (18)	N1—C4—H4	114.3 (13)
			
C4—N1—C1—N3	−179.98 (18)	C1—N2—C2—C3	0.1 (3)
C4—N1—C1—N2	−1.2 (3)	N2—C2—C3—C4	−0.6 (3)
C2—N2—C1—N3	179.55 (19)	C2—C3—C4—N1	0.2 (3)
C2—N2—C1—N1	0.8 (3)	C1—N1—C4—C3	0.6 (3)

### Hydrogen-bond geometry (Å, °)

**Table:**

*D*—H···*A*	*D*—H	H···*A*	*D*···*A*	*D*—H···*A*
N1—H1···O1^i^	0.87 (1)	1.87 (1)	2.742 (2)	177 (2)
N3—H11···O1	0.86 (1)	1.99 (1)	2.850 (3)	178 (2)
N3—H12···O2^i^	0.85 (1)	2.05 (1)	2.901 (2)	178 (2)

Symmetry codes: (i) *x*, *y*−1, *z*.
